# History of the Obesogen Field: Looking Back to Look Forward

**DOI:** 10.3389/fendo.2019.00014

**Published:** 2019-01-29

**Authors:** Jerrold J. Heindel

**Affiliations:** Program on Endocrine Disruption Strategies, Commonweal, Bolinas, CA, United States

**Keywords:** endocrine disruptor, obesogen, metabolism disruptor, developmental origins of disease, obesity

## Abstract

The Obesogen field developed from two separate scientific research areas, endocrine disruptors and the Developmental Origins of Health and Disease (DOHaD). Endocrine Disrupting Chemicals (EDCs) are exogenous chemicals or mixtures of chemicals that interfere with the action of hormones. Exposure to EDCs during early development (DOHaD) has been shown to increase susceptibility to a variety of diseases including infertility, asthma, breast and prostate cancer, early puberty, susceptibility to infections, heart disease, autoimmune disease, and attention deficit hyperactivity disorder/learning disability. The effects of EDCs on obesity and fat cell development first gained attention around the turn of the twenty-first century. In 2002 Dr. Paula Baillie-Hamilton wrote the first review article focusing on environmental chemicals and obesity. She suggested that the obesity epidemic correlated with the increased production of chemicals after World War II. Baillie-Hamilton identified studies showing that exposures to a variety of chemicals led to weight gain. Shortly after that a commentary on an article showing that nonylphenol would increase fat cell differentiation *in vitro* noted the Baillie-Hamilton article and made the point that perhaps obesity was due in part to exposure to EDCs. In 2006 the field of DOHaD/EDCs and obesity made a giant leap forward when Dr. Bruce Blumberg published a paper showing that tributyltin could lead to weight gain in mice and coined the term obesogen for a chemical that caused weight gain and lead to obesity. In 2011, the NIEHS developed the first funding initiative focused on obesogens. In the following years there have been several workshops focused on obesogens. This paper describes these early days that lead to the obesogen hypotheses and the growth of the field for a decade, leading to its prominence today, and provides some insight into where the field is moving.

## Introduction

It is never easy to determine when a scientific paradigm shift has occurred that will lead to the development of a new scientific field, including the development of the obesogen field. Before moving into the history of obesogens *per se*, it is important to note that data supported the concept before anyone thought that unintentional chemical exposures could make people fat. These data came from the pharmaceutical arena, where certain drugs developed for a specific disease had a side effect of causing weight gain. For instance, many individuals on selective serotonin uptake inhibitors, atypical anti-psychotics, tricyclic antidepressants, and thiazolidines antidiabetics experienced weight gains of 5–10 kg (1). The data are particularly strong for rosiglitazone, used to treat type 2 diabetes, which acts as a selective PPAR gamma receptor agonist; a pathway that is known to stimulate fat cell development (2). Monosodium glutamate, a widely used food additive, has a side effect of being a known activator of brain pathways that cause weight gain in animal models (3). Thus, these studies provided proof of principle that chemicals could interfere with metabolism, resulting in weight gain.

## Endocrine Disruptors

In the case of environmental chemicals and obesity, this field emerged from research on endocrine disruptors and the concept of developmental origins of adult disease, now called developmental origins of health and disease (DOHaD). Endocrine disrupting chemicals (EDCs), for the most part, are chemicals designed for a specific purpose, for example, a pesticide or plasticizer, but they also mimic natural hormone actions in the body leading to increased susceptibility to a variety of diseases. The endocrine disruption field began at a Wingspread Conference in 1991 where the term endocrine disruptor was first coined [reviewed in (4)]. There are now around 1,000 chemicals designated as EDCs (https://endocrinedisruption.org/). EDCs are found in a wide variety of products including pesticides/herbicides/fungicides, flame retardants, surfactants, plastics, sunscreens, cosmetics, and personal care products, etc. [reviewed in (5)]. Exposure to EDCs is ubiquitous, such that exposure occurs from air, dust, water and food via ingestion, inhalation, the skin, and the placenta. Some EDCs have short half-lives in minutes or hours while others are highly persistent with half-lives in years (6). Originally, EDCs were shown to interfere with estrogen, androgen and thyroid hormone signaling (7, 8) resulting in diseases and dysfunctions in reproduction, learning, memory, and behavior.

## Developmental Origins of Health and Disease

The DOHaD field posits that development, when tissues are forming, is a highly orchestrated and coordinated process controlled by altering gene expression to make specific tissues with specific genes and functions (9, 10). Hormones and growth factors control this process. Poor nutrition, stress or EDCs can interfere with this developmental trajectory, leading to functional changes in tissues (altered gene expression, altered cell numbers or locations) leading to increased sensitivity/susceptibility to disease and dysfunction over the lifespan (11).

As scientists studied the role of exposure to EDCs in reproductive diseases (e.g., the DOHaD hypothesis), they discovered that these same EDCs could cause weight gain which led to the development of a new scientific field.

## Serendipity and the Birth of a New Field: Integration of EDC and DOHaD Fields

Serendipity, sometimes called unexpected good luck, involves finding something unexpected, realizing its importance and acting on it. Thus, while the initial discovery might be due to luck, the realization of its importance and acting on it is a key component of science. Serendipity played a key role in the birth of the obesogen field.

The first serendipitous discovery came in 1997 when Paula Baillie-Hamilton, a physician, noted that “After the birth of my second son, I gained a significant amount of weight. And I happened to read an article in a national newspaper on how chemical toxins at today's levels of environmental exposure are damaging animals' health by meddling with their hormones. My academic background and previous experience as a doctor had given me the ability to see the connection between these toxins and that perhaps there was a connection to our growing weight problem.” (12). She serendipitously put two scientific fields together for the first time.

In 2002, she published the first paper that focused on a new hypothesis, that chemical toxins could explain the global obesity epidemic (12). In the article she noted, “Therefore it can be posited that the relatively recent presence of synthetic chemicals in the environment may be a significant causative factor in the current worldwide obesity epidemic. These chemicals may be causing weight gain via toxic effects on the body's natural weight-control mechanisms.”

A figure in this publication showed that the increase in obesity followed by several decades the increase in production of chemicals, thus suggesting a link between the two. It further showed that since the early 1970s there had been numerous publications that showed that environmental chemical exposures to rodents could lead to weight gain. However, these were toxicity studies, and the authors did not consider weight gain as a toxic endpoint. The chemicals that she noted as having the ability to cause weight gain include organochlorine pesticides, carbamates, polychlorinated biphenols, plastics such as phthalates and bisphenol A (BPA), heavy metals and solvents. It should be noted that recent studies have indeed confirmed that exposures to these chemicals during development can lead to weight gain in offspring (13). She also noted, “Researchers have reportedly found a positive association between levels of certain toxic chemicals in the children's and adults body tissues and increased weight in these subjects.” Thus, the Baillie-Hamilton review of the literature led her to develop a hypothesis for a new field, which did not exist: an amazing scientific deduction. Unfortunately, the article was published in the Journal of Alternative and Comparative Medicine and therefore was not initially widely noted and cited.

## Serendipity: The First Publications

Two of the articles noted in the Baillie-Hamilton literature review were published by reproductive biologists who were studying the effects of BPA. The authors serendipitously discovered that developmental exposure to BPA would not only result in reproductive effects but would also lead to weight gain (14, 15). While both publications were focused on the reproductive effects of BPA and noted effects on body weight, the Rubin et al. (15) paper mentioned—in the title—that BPA affected body weight. In 2002, several articles showed that childhood obesity is associated with maternal smoking during pregnancy (16, 17), another unexpected result as smoking led initially to lower birth weight. These data led to a review of the fetal origins of obesity focusing on *in utero* nutrition and later-life obesity (18, 19).

At the turn of the twenty-first century, two research areas were coming together, the first of which was the fetal origins of adult disease (now called DOHaD). DOHaD started with a focus on nutrition and the field of environmental chemicals and disease, which initially focused on reproduction. The discovery that developmental exposure to BPA would lead to weight gain led to the development of a new field: the developmental origins of obesity and the role of EDCs.

Data showing that BPA and nonylphenol could stimulate differentiated 3T3-L1 cells into adipocytes followed these initial studies (20–22). These articles were the first to show that an environmental chemical can cause differentiation into adipocytes. In 2003 a commentary (23) on the nonylphenol manuscript was published in Toxicological Sciences (22). This commentary noted the prior Baillie-Hamilton (12) publication and the developing new research area (the fetal basis of adult disease), showing that nutrition *in utero* and early life can have profound influences on birth weight and on lifelong health. It also noted, “the authors of this article on adipocyte differentiation by nonylphenol opened the door to a potentially very exciting new area of research on the action of estrogenic endocrine-disrupting chemicals: one that has enormous implications for public health.” It asked thought provoking questions: “Will these results extrapolate to the *in vivo* situation in rodents and other animal models? Will humans be sensitive to the *in utero* exposure to environmental estrogens about the development of adipocytes? Will toxicology and environmental health sciences play a key role in addressing the obesity epidemic via a reduction in exposures to environmental chemicals *in utero* and throughout life? Only time will tell, but the door has been opened…” While just a commentary, it alerted toxicologists to the Baillie-Hamilton article and the new area of fetal origins of obesity.

In 2005, Newbold et al. (24) first showed that neonatal exposure to low doses of diethylstilbestrol (DES) would cause obesity as measured both by increased weight and increased fat in females. The picture, published in 2009 (25) on the effect of neonatal DES exposure on weight gain in adults has, in today's vernacular, gone viral as it has been shown as a key example of an estrogenic chemical and obesity many times.

## Epidemiology Studies on EDCs and Obesity

Smoking during pregnancy is a proof of principle for the ability of chemicals to cause increased risk for overweight in childhood. The first publications showing that smoking during pregnancy would increase weight gain in children were published in 2002 and 2003, predating an obesogen field. In 2008, Oken et al. conducted a meta-analysis of 14 epidemiology studies that showed a strong association between smoking during pregnancy and weight gain in the children (18). Smoking throughout pregnancy has the largest effect on childhood weight, and there was a dose response with more smoking leading to a larger effect on the children's weight. By 2013, there were over 30 separate epidemiology publications that all showed the same effect; increased weight gain in children from the mother's smoking during pregnancy, something unheard of in the epidemiology literature (26). These results were confirmed in human studies that examined nicotine administration to pregnant mothers [reviewed in (18)] and in animal studies showing that nicotine increased body weight and fat disposition, adipocyte size and gene markers of adipogenesis as well as decreases physical activity (27, 28). Even today, smoking during pregnancy represents the strongest data point supporting both the importance of development as a sensitive period for weight gain later in life and for nicotine being an endocrine disruptor. Interestingly, in this instance, epidemiological data was the first to show the association between smoking and weight gain, and the data appeared before anyone thought that chemicals (EDCs) could cause weight gain.

Beth Gladen, who had a history of studying polychlorinated biphenyls (PCBs) and dioxins in breast milk and pregnancy outcomes in humans, appears to have published the first epidemiology study on EDCs and obesity (29). They showed that girls born from mothers with the highest PCB and DDE (metabolite of DDT) exposures during pregnancy were heavier for their heights than other girls. In 2004, this same group showed that prenatal exposure to DDT was not associated with BMI (30). Despite this dubious start in the early 2000s, by 2014, 33 publications examined either overweight, BMI or general growth. Twenty-two publications examined organochlorine pesticides (DDT, hexachlorobenzene), and seven examined PCBs. There were also publications that assessed the effects of polyaromatic hydrocarbons, perfluorooctanoic acid(PFOA)/perfluorooctane sulfonate (PFOS), BPA and dioxins (31). The first comprehensive review of childhood obesity and environmental chemicals appeared in 2011(32).

## Serendipity and the Obesogen Hypothesis

What is in a name? A lot. In 2006, the focus of the Blumberg lab was on orphan receptors, with an emphasis on PPARγ and RXR. They noted that tributyltin which activates PPARγ and RXR in mollusks also increased fat in this model. This serendipitous discovery changed the direction of the lab to a focus on the ability of tributyltin to cause weight gain in mice and differentiation of 3T3-L1 cells into lipid accumulating adipocytes. These data lead Grun and Blumberg to publish their classic paper which introduced the term “environmental obesogen.” They defined an obesogen as molecules that inappropriately regulates lipid metabolism and adipogenesis to promote obesity (33). The obesogen hypothesis stimulated the field in general by giving a catchy, easy-to-remember name to a subset of EDCs that can stimulate obesity. They noted, “the existence of chemical obesogens in and of themselves suggests that the prevailing paradigm, which holds that diet and decreased physical activity alone are the causative triggers for the burgeoning epidemic of obesity, should be reassessed.”

As an indication of the growth of this new field, or perhaps to stimulate this new field, several reviews appeared in 2007, 2008, and 2009 (7, 25, 34–39), and this trend continues today (13, 40–44).

## Workshops

The first workshop that focused on the role of developmental exposures to environmental chemicals and obesity was held at Duke University and sponsored by the National Institute of Environmental Health Sciences (NIEHS) and the Duke University Integrated Toxicology Program in 2004 (45). The goal of the workshop was to highlight the available data as a proof-of-concept in animals and to stimulate interest in this research area. The workshop focused on *in utero* nutrition and obesity, basic biology of fat cells and their control and several talks related to the effects of developmental exposure to environmental chemicals and later onset of obesity.

In 2011, the National Toxicology Program at the National Institute of Environmental Health Sciences held a workshop, “Role of Environmental Chemicals in the Development of Diabetes and Obesity.” At this workshop, a diverse group of scientists was asked to evaluate the current literature for consistency and biological plausibility (46). The strongest conclusion from the workshop was that nicotine is an obesogen, followed by the animal and *in vitro* data for tributyltin being an obesogen and the human data on some persistent organic pollutants and diabetes. The data on BPA were considered suggestive of effects on glucose homeostasis, insulin release and adipogenesis (26, 46–48). The workshop proceedings identified research gaps including more research on type 1 diabetes, lack of human studies, more studies on the basic biology of adipocytes, and a need for more information on the biology that controls body weight and metabolic set points that change with life stage. There was also a discussion of how high throughput screening could provide valuable information about chemicals, pathways, and endpoints.

The first International workshops on obesity, “Obesity and Environmental Contaminants,” were held in Uppsala, Sweden, in 2013 and 2015. A consensus statement was published as a result of the 2nd International Workshop on Obesity and Environmental Contamination, held in 2015 (49). It discussed several actions that could be taken to restrict the use of potentially harmful contaminants on metabolism.

In 2014, a small group of scientists met in Parma, Italy, to address how to help move the obesogen field forward. This was the first meeting to focus not just on obesogens and obesity, but to expand the focus to metabolic disruptors, chemicals that can cause obesity, diabetes, and fatty liver disease including metabolic syndrome. This workshop resulted in two key publications: the Parma Consensus statement (50) and a review of metabolism and the chemicals that have been shown to disrupt metabolism (13).

## Funding and Scientific Support

Funding for the research area of environmental chemicals and obesity has come mainly from the NIEHS in the USA. Indeed, from the first publications noted above (14, 15), essentially all the publications related to *in vitro* and *in vivo* obesogen animal models and human studies were supported by NIEHS. Nonetheless, it took until 2011 for the NIEHS to release a specific funding announcement that focused on the role of environmental chemicals and obesity and diabetes. This announcement led to the funding of more than a dozen animal and human birth cohort studies which stimulated research and interest in the field over 3 years. This has been the only major funding focused specifically on understanding the role of EDCs in the obesity epidemic.

There is no EDC Society to specifically support and coordinate EDC, and therefore obesogen, research. However, in 2005 the Endocrine Society committed itself to EDCs as an important issue (http://press.endocrine.org/doi/10.1210/en.2005-1367) (51). In 2009, the Endocrine Society developed a position statement on EDCs followed by a Scientific Statement on EDCS (52), which established EDC research as a policy priority backed by a strong foundation of science. EDC-2. The Endocrine Society's Second Scientific Statement on Endocrine-Disrupting Chemicals was published in 2015 and contained a focus on obesogens (5). Thus, the Endocrine Society can be considered a home for obesogen research.

## Summary of the First Decade

**Figure 1:**
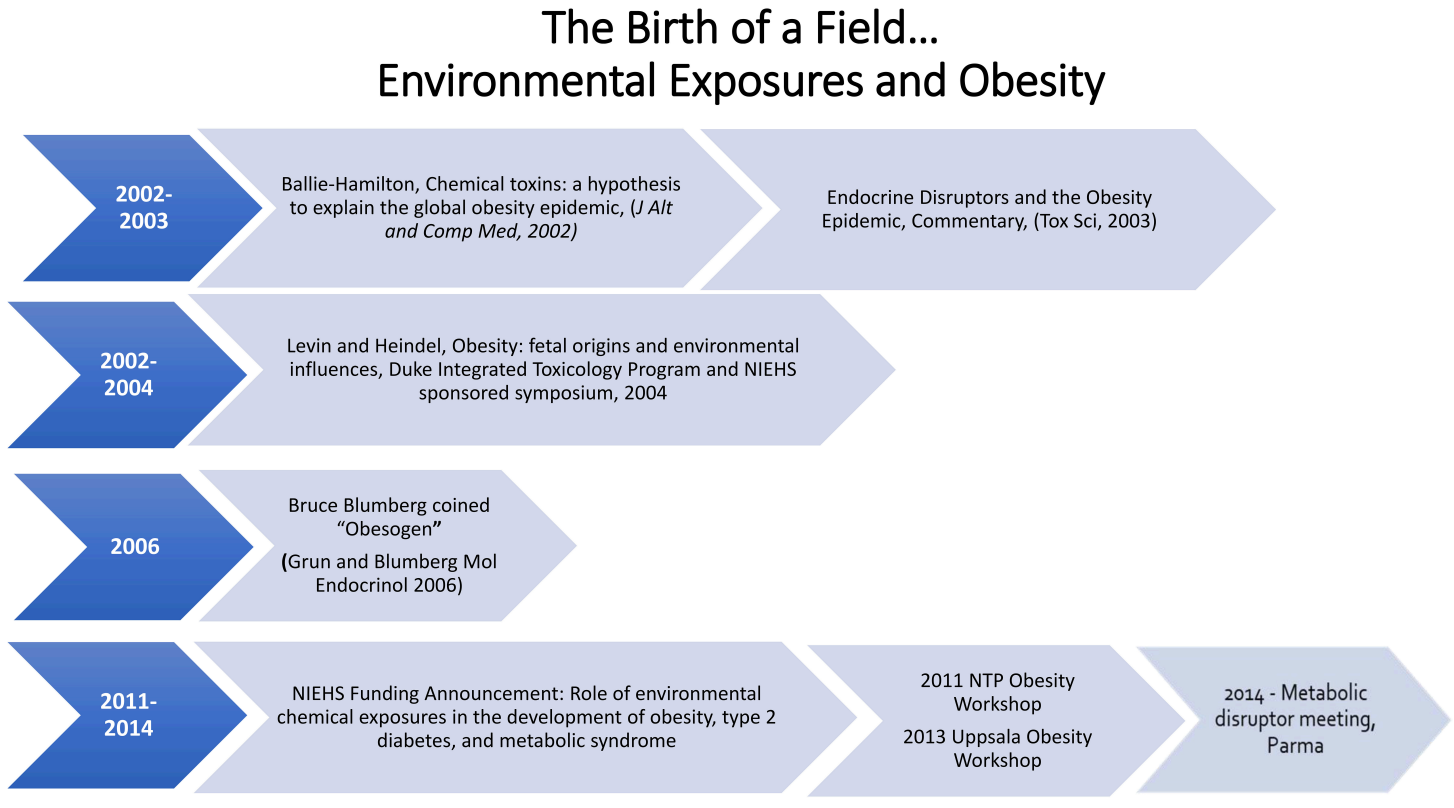
Timeline of obesogen field.

The first 10 years of the obesogen hypothesis has led to research which has identified chemicals of concern and developmental windows of sensitivity (*in utero* and neonatal). In addition, publications showed sexually dimorphic differences in the effects of obesogens: developmental exposure to DES or BPA resulted in obesity only in the female offspring (24, 53). There are now numerous chemicals that can be designated as obesogens (13, 40, 54, 55). See Box 1 and Figure 1.

Box 1 **Examples of obesogenic chemicals and their sources.**

**Antimicrobial:** Triclosan, Paraben(s).

**Biogenic compounds:** Isoflavones (genistein, daidzein), Nicotine, Permethrins.

**Byproducts/intermediate reactants:** Dioxin, Nonylphenol, Acrylamide, Bisphenol A(BPA), Perfluorooctanoic acid (PFOA), Tributyltin, Benzo(a)pyrene.

**Flame retardants:** Tetrabromobisphenol A (TBBPA), Polybrominated diphenyl ethers (PBDE), Firemaster 550.

**Food additives and contact materials:** Monosodium glutamate, Tributyltin, High fructose corn syrup, nonmetabolizable sugars.

**Household product ingredient:** Acrylamide, di(2-ethylhexyl) phthalate (DEHP), Tributyltin, Triclosan, Bisphenol A diglycidyl ether (BADGE), Parabens.

**Industrial additive:** di (2 ethylhexyl) phthalate (DEHP), dibutyl phthalate (DBP), Tributyltin, Persistent Organic Pollutants (POPs).

**Medical/veterinary research:** Acrylamide, Bisphenol A diglycidyl ether (BADGE), Butyl benzyl phthalate (BBzP), Neonicotinoid Insecticide -imidacloprid, Permethrins, Tetrabromobisphenol A (TBBPA), Tributyltin, Diethylstilbestrol, Thiazolinedione antidiabetics (rosiglitazone), Tricyclic antidepressants (amitrriiptyline, Miratazapine), Selective serotonin uptake inhibitors.

**Metabolite/degradate:** Butyl benzl phthalate (metabolite of BBzP), Mono-(2-ethylhexyl) phthalate (metabolite of DEHP), Butyl phthalate (metabolite of DBP), o, p'DDE (metabolite of DDT).

**Metal/metalurgy:** Lead, Arsenic, Cadmium.

**Personal care products/cosmetic ingredients:** Perfluorooctanoic acid (PFOA), Perfluroooctane sulfonate (PFOS), butyl paraben, methyl paraben, di(2-ethylhexyl) Phthalate (DEHP), dibutyl phthalate (DBP), triclosan.

**Pesticide/fungicide and ingredient**: di(2-ethylhexyl)phthalate (DEHP), Dibutyl phthalate (DBP), Methyl paraben, Perfluorooctane sulfonic acid (PFOA), triclosan, Parathion, Organophosphate Pesticides (Diazinon, Chlorpyrifos) Imidacloprid, Triflumizole, Zoxamide, Quinoxyfen, Fludioxonil, Organochlorine Pesticides (Dichlorophenyltrichlorethane (DDT), Hexachlorobenzene (HCB), Lindane), Pyrethroid Pesticides (Permethrin, Deltamethrin), Phenylpyrazole Pesticide (Fipronil), Fungicide (Pyraclostrobin).

**Plastic/rubber**: Octyl phenol, acrylamide, Bisphenol A(BPA), Bisphenol A diglycidyl ether (BADGE), Bisphenol S, di(2-ethylhexyl) phthalate (DEHP), Perfluorooctanoic acid (PFOA), Tributyl tin, Triclosan.

**Solvent:** Dibutyl phthalate (DBP).

**Air pollutants:** Poly Aromatic Hydrocarbons (PAH), PM 2.5.

Note that some chemicals occur in multiple classes indicating several venues for exposure. This list is derived from review articles noted in this review as well as the listing of EDCs found on The Endocrine Disruption Exchange website: https://endocrinedisruption.org/

The animal and human data developed in the first decade have led to the following conclusions outlined in Heindel (50):

- Susceptibility to obesity is at least in part “programmed” *in utero* and early postnatal life by exposures to environmental stressors including obesogens. This developmental programming can be considered to alter the “set point” or sensitivity to develop obesity. Indeed, it is likely that obesogens, due to their actions on programming metabolism, alter the amount of food needed to result in weight gain and the amount of exercise needed to lose weight.
- Programming may alter the number, size and function of fat cells, as well as effects on the brain appetite and/or satiety centers, control of the GI tract, muscle, pancreas, and liver, leading to altered sensitivity for gaining weight.
- It is likely that we are underestimating the importance of obesogens because of the focus of current research on a single or small subset of chemicals at a time, during limited windows of sensitivity, in single tissues and focusing on endpoints related to only one metabolic disease.

## The Obesogen Field Today

In the last few years there have been two major advances in the field: a focus on transgenerational inheritance by obesogens and a change from a focus on obesogens to metabolism disruptors.

Transgenerational epigenetic inheritance due to exposure to environmental chemicals was first shown in 2005 (56) and it focused on reproductive endpoints. The first report of obesity being inherited transgenerationally by environmental chemicals appeared in 2013 (57). There are currently publications linking ancestral exposure to dichlopdiphenyltrichloroethane (DDT) (57), a combination of BPA, diethyl hexyl phthalate and dibutyl phthalate (58) tributyltin (59, 60) and jet fuel (61) to obesity. These transgenerational inheritance studies are the most disturbing as they show that the effects of obesogen exposure during pregnancy may be apparent in future generations. Perhaps some of the global obesity epidemics noted today are due to exposures to past generations in addition to current exposures.

While the term obesogen is still valid, it soon became apparent that some obesogens had activity at other tissues leading to type 2 diabetes, fatty liver and indeed metabolic syndrome. The first publication that changed the focus from obesogens *per se* to metabolic disruption and metabolic disruptors appeared in 2011 (62). This expansion from obesogen to metabolic disruptors was developed further in 2015 (13, 50) and again in 2017 (13, 55) in reviews that discussed moving to the new term metabolism disrupting chemicals (MDCs) for chemicals that cause not just weight gain but also type 2 diabetes and non-alcoholic fatty liver disease.

## Future Directions

As the obesogen field moves into its second decade, an iceberg may be a good representation of the state of the science. The current data are just the tip of the iceberg, the part that shows above the water. The part of the iceberg under the water is likely to be larger, indicating that there remain many questions to answer including, how many obesogens are there, what are their molecular targets, what are the critical windows of exposure, how many windows of exposure are there and how do they interact?

Indeed, the field is still quite small, with only a few dedicated researchers focused on understanding the role of environmental chemicals in the epidemics of obesity, diabetes, and liver diseases. Thus, the first and main objective must be to expand the cadre of researchers. Just as the first researchers came from other fields, it is imperative that animal, epidemiology and clinical researchers from other fields use their expertise to help understand the role of environment in these metabolic diseases. The obesogen field, along with the focus on metabolism disruptors, has no society or specific training or coordination. The development of some oversight and coordination would be helpful to the science, outreach to clinicians and public as well as interaction with policy makers. Finally, since it is now clear that developmental exposures to obesogens/MDCs play a role in the obesity epidemic, there needs to be a concerted effort to focus on prevention by reducing exposures during windows of susceptibility and across the lifespan. Making an effort to prevent disease is always the best strategy!

Also, the field would be improved by

- Development of an integrated conceptual approach linking animal studies and endpoints with longitudinal human cohort studies.
- Development of studies that focus on the ability of obesogens/MDCs to not cause obesity or other metabolic diseases *per se*, but to show how altering the “set point” or sensitivity for gaining weight and the ability to lose weight, can alter the amount of food needed to gain or lose weight or the amount of exercise needed to lose weight.
- Assessment of multiple chemicals, mixture studies, and integration of environmental chemical studies with other stressors including stress, drugs nutrition, and infections.
- Identification of windows of sensitivity, how many are there, what mechanisms underly a window of sensitivity, how multiple windows interact across the lifespan and generations.
- Focus on environmentally relevant doses of chemicals when assessing their obesogenic effects.
- Focus on assessment of both males and females as obesogenic effects tend to be sexually dimorphic.
- Assessment of the interaction of diet including high-fat and/or high carbohydrate diets with obesogen exposures, since diet can modulate obesogen activity.
- Focus on transgenerational inheritance. Will all obesogens/MDCs be transgenerational obesogens or just a subset; if a subset, what are the characteristics that allow transgenerational effects?
- Development and validation of *in vivo* and *in vitro* screens to detect and prioritize obesogens/MDCs.
- Work with clinicians and clinical societies to improve their understanding of the importance of environmental chemicals in metabolic disorders.

## Author Contributions

The author confirms being the sole contributor of this work and has approved it for publication.

### Conflict of Interest Statement

The author declares that the research was conducted in the absence of any commercial or financial relationships that could be construed as a potential conflict of interest.
